# A 0.6-µW Chopper Amplifier Using a Noise-Efficient DC Servo Loop and Squeezed-Inverter Stage for Power-Efficient Biopotential Sensing

**DOI:** 10.3390/s20072059

**Published:** 2020-04-06

**Authors:** Xuan Thanh Pham, Ngoc Tan Nguyen, Van Truong Nguyen, Jong-Wook Lee

**Affiliations:** School of Electronics and Information, Information and Communication System-on-chip (SoC) Research Center, Kyung Hee University, Yongin 17104, Korea; thanhpham@khu.ac.kr (X.T.P.); nguyenngoctanbk10@gmail.com (N.T.N.); nguyenvantruong.bk@gmail.com (V.T.N.)

**Keywords:** ultra-low power, instrumentation amplifier, body control, electrode offset, dc servo loop, input-referred noise

## Abstract

To realize an ultra-low-power and low-noise instrumentation amplifier (IA) for neural and biopotential signal sensing, we investigate two design techniques. The first technique uses a noise-efficient DC servo loop (DSL), which has been shown to be a high noise contributor. The proposed approach offers several advantages: (i) both the electrode offset and the input offset are rejected, (ii) a large capacitor is not needed in the DSL, (iii) by removing the charge dividing effect, the input-referred noise (IRN) is reduced, (iv) the noise from the DSL is further reduced by the gain of the first stage and by the transconductance ratio, and (v) the proposed DSL allows interfacing with a squeezed-inverter (SQI) stage. The proposed technique reduces the noise from the DSL to 12.5% of the overall noise. The second technique is to optimize noise performance using an SQI stage. Because the SQI stage is biased at a saturation limit of 2*V*_DSAT_, the bias current can be increased to reduce noise while maintaining low power consumption. The challenge of handling the mismatch in the SQI stage is addressed using a shared common-mode feedback (CMFB) loop, which achieves a common-mode rejection ratio (CMRR) of 105 dB. Using the proposed technique, a capacitively-coupled chopper instrumentation amplifier (CCIA) was fabricated using a 0.18-µm CMOS process. The measured result of the CCIA shows a relatively low noise density of 88 nV/rtHz and an integrated noise of 1.5 µV_rms_. These results correspond to a favorable noise efficiency factor (NEF) of 5.9 and a power efficiency factor (PEF) of 11.4.

## 1. Introduction

Recently, there is a growing interest in wearable, portable, and personal health monitoring. By detecting abnormal health conditions during daily monitoring, this approach provides a new method of preventive healthcare. Monitoring human biopotential and neural signals is also important for early diagnosis and medical treatment [1]. Concerning the biopotential monitoring applications, sensors and their interfaces providing high-quality signals are of great importance. Besides, these sensor devices demand both long operating time and a compact form factor. A battery is widely used, however, it requires frequent battery recharging or replacement, and there is a limit in its size for some applications such as implantable sensors. To achieve a compact form factor by reducing the volume of the battery, low power consumption is in great demand for wearable and portable sensor devices.

Signals from humans have an amplitude of around 1 mV for an electrocardiogram (ECG) and from 10 to 100 µV for an electroencephalogram (EEG) over a frequency band from 0.5 to 150 Hz [2]. The local field potential (LFP) has a typical amplitude of 1 mV over 1 to 200 Hz. These low-frequency signals must first be amplified before any signal processing can be applied. One issue with amplification is the overlap of these signals with 1/*f* noise. To mitigate the effect of 1/*f* noise, a chopping technique can be applied for instrumentation amplifiers (IAs) [3,4,5,6,7,8,9,10,11,12]. Another issue is the electrode offset voltage *V*_EOS_ generated at the tissue-electrode interface by electrochemical effects. To reject *V*_EOS_, a DC servo loop (DSL) has been used in a capacitively-coupled chopper instrumentation amplifier (CCIA) [3]. This approach has the advantage of removing bulky external capacitors. However, the DSL achieves the *V*_EOS_ rejection by increased input-referred noise (IRN). The IRN $\bar{V_{n,\mathrm{in}}^{2}}$ of a CCIA can be expressed as [3]

(1) $$\bar{V_{n,\mathrm{in}}^{2}}={\left(\frac{C_{\mathrm{in}}+C_{\mathrm{fb}}+C_{\mathrm{hp}}+C_{P}}{C_{\mathrm{in}}}\right)}^{2}\bar{V_{n,\mathrm{in},\mathrm{Gm}}^{2}}$$

where $\bar{V_{n,\mathrm{in},\mathrm{Gm}}^{2}}$ represents the input-referred noise of a transconductor. The *C*_in_, *C*_fb_, and *C*_P_ are the input, feedback, and parasitic capacitors that are connected to the input of the CCIA, respectively. The *C*_hp_ is the capacitor in the DSL which is used to create a high-pass corner to reject *V*_EOS_. When a large *C*_hp_ is used, the result (1) shows that it increases the IRN by charge dividing, causing the DSL to be a high noise contributor; previous studies often neglected this important issue. For example, the IRN increases from 0.7 to 6.7 µV_rms_ [4] and from 2.8 to 4.7 µV_rms_ [6] when the DSL is enabled. Thus, in these cases, the DSL contributes 89.5% [4] and 40.4% [6] of the overall noise. The increased noise significantly degrades both the noise efficiency factor (NEF) [7] and the power efficiency factor (PEF) [10].

Several methods have been proposed to improve the DSL. In [5], a digitally-assisted foreground calibration is used to allow the DSL to handle residual offset. In [7], a dual DSL which consists of coarse and fine DSLs reduces the value of *C*_hp_ from 670 to 100 fF. In [8], the output of a DSL is connected to the cascode branch of a transconductor to mitigate the charge dividing effect. Nevertheless, these CCIAs consume 3.48 µW [7] and 2.13 µW [8], which results in relatively high PEFs of 18.3 and 10.5 (over a 10-kHz bandwidth), respectively. The results indicate that previous work suffers from high noise contribution from the DSL and achieves a relatively low noise-power efficiency.

In this paper, we investigate two design techniques to realize a 0.6-μW chopper amplifier with a PEF of 11.4 over a 200-Hz bandwidth. The first technique optimizes noise performance using a squeezed-inverter (SQI) stage. Because the SQI stage allows for the reduction of the supply voltage to a saturation limit of 2*V*_DSAT_, its bias current can be increased to reduce noise. The second technique is to reduce the relatively high noise from the DSL. Unlike conventional DSLs, which are connected to the input of the CCIA through *C*_hp_, we apply the output of the DSL to the body of a transconductor. The proposed approach not only removes the charge dividing effect but also reduces the noise by the transconductance ratio and the open-loop gain. Furthermore, this approach solves the problem of interfacing the DSL to the SQI stage, which has a different supply voltage. Using this approach, the noise contribution of the DSL is reduced to 12.5%. The fabricated CCIA achieves a relatively low noise density of 88 nV/rtHz with an integrated noise of 1.5 μV_rms_. The result corresponds to a favorable NEF of 5.9 and a PEF of 11.4 by consuming only 0.68 μW, demonstrating a power-efficient low-noise amplifier.

## 2. Design

**Figure 1:**
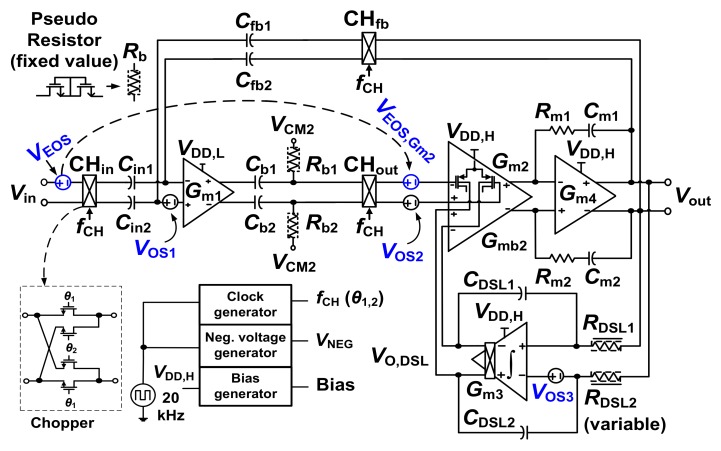
Schematic of the proposed capacitively-coupled chopper instrumentation amplifier (CCIA) using body-controlled DC servo loop (DSL). *C*_in1,2_ = 4 pF, *C*_fb1,2_ = 40 fF, *C*_b1,2_ = 3 pF, *C*_m1,2_ = 4 pF, *R*_m1,2_ = 4 MΩ, and *C*_DSL1,2_ = 5 pF.

Figure 1 shows the schematic of the proposed CCIA. The input transconductor *G*_m1_ is realized using an SQI stage biased at *V*_DD,L_ = 0.2 V. The transconductors *G*_m2_, *G*_m3_, and *G*_m4_ are folded-cascode, two-stage opamp, and common source stages biased at *V*_DD,H_ = 0.8 V, respectively. Transconductor *G*_m3_ is used as the integrator in the DSL. We consider the input offset voltages *V*_OS_*_i_* (*i* = 1 to 3) for *G*_m_*_i_*. The input *V*_in_ is up-modulated to chopping frequency *f*_CH_ by the chopper CH_in_, then down-modulated to baseband by CH_out_. The common-mode (CM) voltage *V*_CM2_ = *V*_DD,H_/2, which bypasses the chopper CH_out_, is used to bias *G*_m2_ through pseudo-resistors *R*_b1,2_. A Miller capacitor *C*_m1,2_ is used for stability. The mid-band gain of the CCIA is defined by input capacitor *C*_in1,2_ and feedback capacitor *C*_fb1.2_. The current consumptions of *G*_m1_, *G*_m2_, *G*_m3_, and *G*_m4_ are 1.61 µA, 60 nA, 210 nA, and 80 nA, respectively.

Although the SQI stage provides low noise operation, interfacing it with the DSL poses a challenge. This is because the input range of the SQI stage is limited by *V*_DD,L_ = 0.2 V, while the DSL senses the output *V*_out_ with a wide swing. We believe that this is one reason why previous studies do not implement a DSL [10]. To solve this problem, we modify the conventional DSL by connecting the output *V*_O,DSL_ of the DSL to *G*_m2_ using the body terminal. We note that this approach is different from the previous approach wherein the output of the DSL is connected to the virtual ground node of the input transconductor through *C*_hp_ [3,4,6]. The proposed approach offers several advantages: (1) because the proposed DSL uses *G*_m2_ instead of *C*_hp_, the charge dividing effect is removed and noise from the DSL is reduced, (2) the noise from DSL is further reduced by the open-loop voltage gain *A*_V1_ of *G*_m1_ as well as by the square of the transconductance ratio, and (3) by rejecting both *V*_EOS_ and *V*_OS2_, output offset is suppressed.

**Figure 2:**
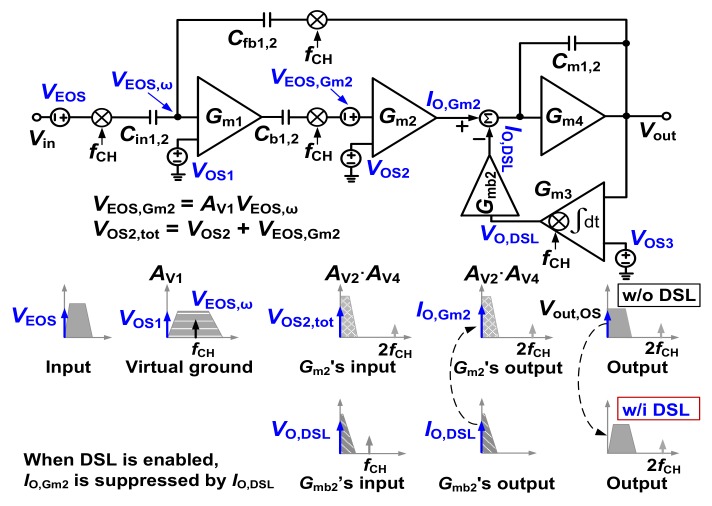
A simplified model of the proposed CCIA.

**Figure 3:**
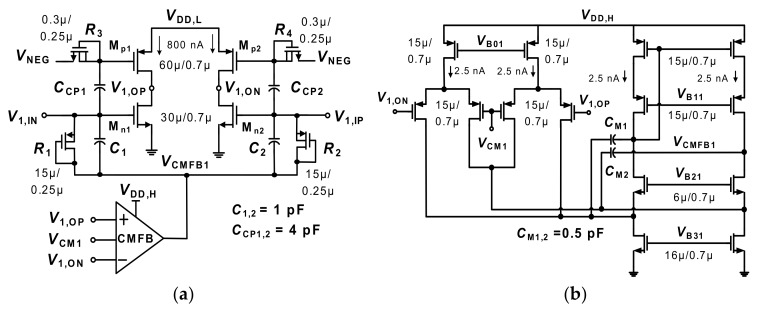
(**a**) Schematic of the squeezed-inverter stage using the shared common-mode feedback (CMFB). *V*_CM1_ = 0.1 V, *V*_NEG_ = −0.18 V, and *V*_CMFB1_ = 0.098 V (nominal value). (**b**) Schematic of the proposed CMFB circuit. *V*_B01_ = 0.57 V, *V*_B11_ = 0.28 V, *V*_B21_ = 0.49 V, and *V*_B31_ = 0.23 V.

Figure 2 shows the simplified model of the proposed CCIA. Offset voltage *V*_OS1_ creates an output ripple due to finite amplifier bandwidth. The amplitude of the output ripple can be expressed as *V*_out__,__r__ipple_ = (*V*_OS1_*A*_V1_*G*_m2_)/(2*C*_m1,2_*f*_CH_) [4]. To suppress this ripple, we use capacitors *C*_b1,2_ in front of CH_out_. Because *V*_OS1_ is blocked by *C*_b1,2_, the residual ripple appearing at *f*_CH_ can be neglected. Both *V*_OS2_ and *V*_EOS_ create output offset *V*_out,OS_ at the output. The rejection of *V*_EOS_ and *V*_OS2_ is explained as follows: When *V*_EOS_ is up-modulated to *f*_CH_, it is partially suppressed by *C*_fb1,2_ at the virtual input node of *G*_m1_. The residual offset *V*_EOS,ω_ existing at *f*_CH_ can be expressed as *V*_EOS,ω_ = *V*_EOS_ *C*_in1,2_/(*A*_V_*C*_fb1.2_), where *A*_V_ is the overall open-loop voltage gain of the amplifier. This residual offset is amplified by *A*_V1_. Simulation results show that *A*_V1_ is 29 dB with a low-pass corner of 954 kHz. Additionally, a high-pass corner frequency of 1 Hz is created by *C*_in1,2_ and bias resistor *R*_1,2_ inside *G*_m1_ (See Figure 3). Then, *V*_EOS,ω_ is down-converted by CH_out_ to create an offset voltage *V*_EOS,Gm2_ = *A*_V1_*V*_EOS,ω_ at the input of *G*_m2_. We observe the sum of offset voltages, *V*_OS2,tot_ = *V*_OS2_ + *V*_EOS,Gm2_, at the input of *G*_m2_. Transconductors *G*_m2_ and *G*_m4_ have a low-pass characteristic with a 3-dB frequency of about 10 Hz, and *V*_OS2,tot_ generates offset current *I*_O,Gm2_ at the output of *G*_m2_. The offset current is integrated by *G*_m4_, which creates the output offset *V*_out,OS_. This is sensed by the DSL, then *V*_O,DSL_ is applied to the body of the differential pair of *G*_m2_. The generated current *I*_O,DSL_ = *G*_mb2_*V*_O,DSL_ compensates *I*_O,Gm2_. The DSL continues integrating, and *V*_out,OS_ is suppressed by the amount 1/*LG*(s), where the loop gain *LG* can be expressed as *LG*(s) = *g*_mb1,2_/(*s*^2^*C*_m1,2_*R*_DSL1,2_ *C*_DSL1,2_).

The selection of *f*_CH_ involves considering the various tradeoff between input impedance, output ripple, and residual offset. *V*_out__,__r__ipple_ can be reduced by increasing *f*_CH_. One drawback of increasing *f*_CH_ is that it reduces the input impedance *Z*_i__n_. Besides, there is greater charge injection and clock feed-through during the switching of the chopper [13]. To determine suitable *f*_CH_, we perform periodic steady-state (PSS) and periodic noise analysis (PNOISE) simulations. Considering the tradeoff and the amplifier bandwidth, we select *f*_CH_ = 10 kHz.

## 3. Circuit Implementation

Figure 3a shows a schematic of the *G*_m1_ implemented using an SQI stage [10] modified to improve the common-mode rejection ratio (CMRR). The transistors in the SQI stage are biased in the subthreshold region using *V*_DD,L_ = 0.2 V. The IRN of the *G*_m1_ can be expressed as

(2) $$\bar{V_{n,\mathrm{in},\mathrm{Gm}1}^{2}}=\frac{8kT}{g_{m,n}+g_{m,p}}≅\frac{4kTnU_{\mathrm{th}}}{I_{\mathrm{DC}}}$$

where *I*_DC_ = 800 nA is the bias current, *g*_m,n_ and *g*_m,p_ are the transconductance of M_n1_ and M_p1_, respectively, *U*_th_ = 26 mV is the thermal voltage, and *n* = 1.5 is the subthreshold factor [9]. The SQI stage reduces the noise by increasing *I*_DC_. Because the supply voltage is reduced to a saturation limit of 2*V*_DSAT_~0.2 V, both low noise and low power operation can be achieved.

To generate *I*_DC_, bias voltages beyond supply rails are used for M_n1_ and M_p1_. The bias voltage for M_n1_ is pushed above the supply rail by using a common-mode feedback (CMFB) loop. The bias voltage *V*_NEG_ for M_p1_ is pushed below the ground by using a negative voltage generator, which is applied to the gate of M_p1_ through a pseudo-resistor *R*_3,4_. Because the transistors work in the subthreshold region without a tail current source, balancing the bias current for the input pair is challenging. To address this, we use a shared CMFB loop. Figure 3b shows the schematic of the CMFB circuit for the SQI stage. It monitors the CM voltage of outputs *V*_1,ON_ and *V*_1,OP_. Then, the output *V*_CMFB1_ of the CMFB circuit is applied to the gate of M_n1,,2_ through pseudo-resistors *R*_1,2_. Because any change in *V*_CMFB1_ affects the input pair by the same amount, this approach provides balanced bias currents for the SQI stage.

**Figure 4:**
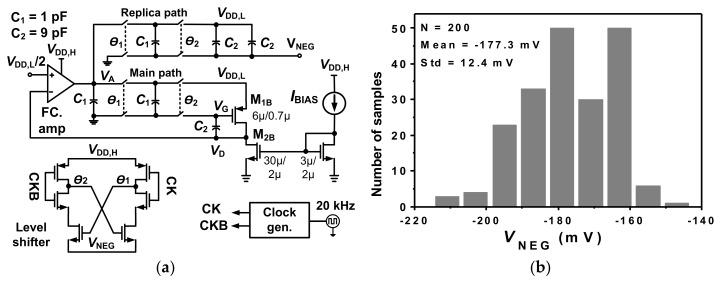
(**a**) Schematic of the negative voltage generator, (**b**) statistical distribution of *V*_NEG_.

Figure 4a shows the schematic of the negative voltage generator. It consists of a 1/10-scaled current replica, two switched-capacitor (SC) paths, a level shifter, and a folded-cascode (FC) amplifier. The SC network consists of the main path and a low noise replica. The FC amplifier and the main SC path generate the bias voltage *V*_G_ for M_1B_ by regulating *V*_D_ to *V*_DD,L_/2. The replica path is responsible for copying *V*_G_ to generate *V*_NEG_. The current mirror defines an 80 nA through M_1B_, which is the 1/10-scaled current of M_p1,2_. The negative voltage generator draws 18 nA from V_DD,H_ and 80 nA from V_DD,L_. Figure 4b shows the statistical distribution of *V*_NEG_ obtained from Monte Carlo simulations. The result shows an average value of −177.3 mV with a standard deviation of 12.4 mV.

**Figure 5:**
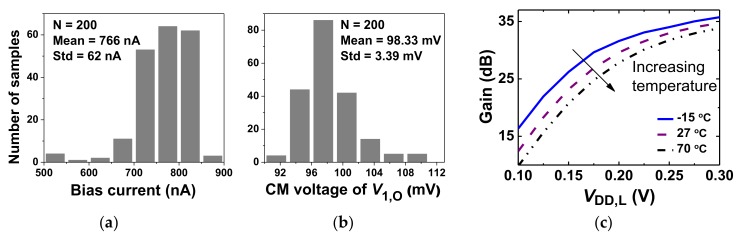
Monte Carlo simulation results for the (**a**) bias current, (**b**) output common-mode (CM) voltage of the squeezed-inverter (SQI) stage. (**c**) simulated gain of the SQI stage depending on *V*_DD,L_ and temperature.

Figure 5a,b shows the statistical distributions of the bias current and the output CM voltage obtained from 200 Monte Carlo simulations. Both random mismatch and process variations are considered. The result shows an average bias current of 766 nA with a standard deviation of 62 nA. The output CM voltage shows an average value of 98.3 mV with a standard deviation of 3.4 mV. Compared to previous work which uses two separate CMFB loops [10], the proposed approach increases the CMRR from 85 to 105 dB. This indicates that the proposed shared CMFB loop is effective in improving CMRR. Figure 5c shows the gain of the SQI stage depending on temperatures as a function of *V*_DD,L_. Because the transistors are biased in the subthreshold region, the increased threshold voltage with temperature reduces the gain [10]. We note that the SQI stage still provides a gain >20 dB when *V*_DD,L_ is reduced to 0.15 V at 70 °C. At room temperature, the SQI stage achieves a gain of 29 dB with *V*_DD,L_ = 0.2 V.

**Figure 6:**
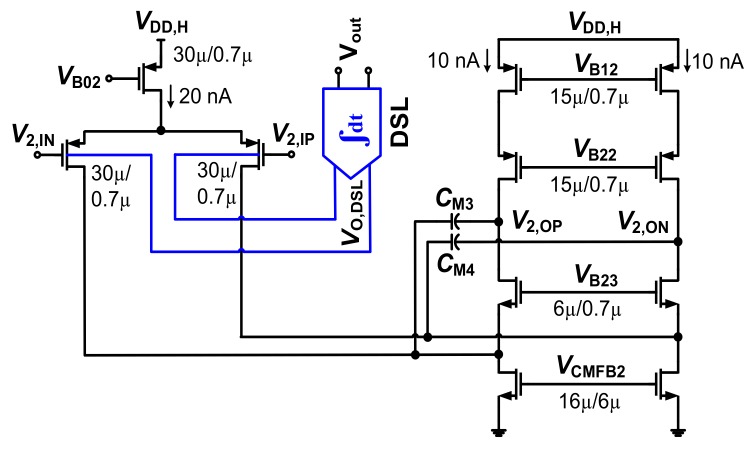
Schematic of transconductor *G*_m2_ with body-controlled DSL. *C*_M3,4_ = 0.5 pF. *V*_B02_ = *V*_B12_ = 0.52 V, *V*_B22_ = 0.18 V, *V*_B23_ = 0.6 V, and *V*_CMFB2_ = 0.28 V (nominal value).

**Figure 7:**
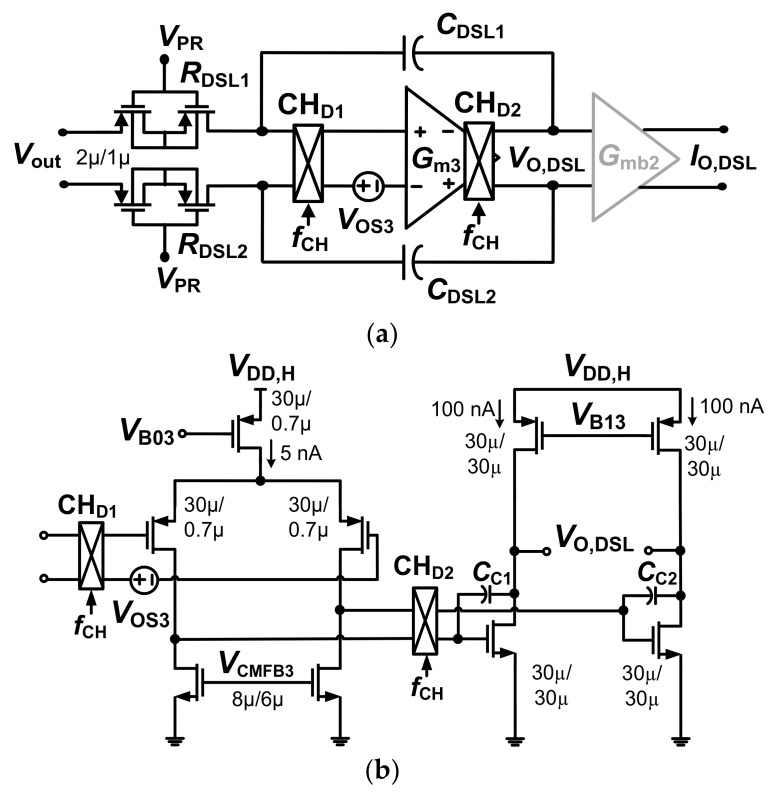
(**a**) schematic of the DSL and (**b**) schematic of the two-stage opamp *G*_m3_. *C*_DSL1,2_ = 5 pF, *C*_C1,2_ = 0.5 pF. *V*_B03_ = 0.57 V, *V*_B13_ = 0.34 V, and *V*_CMFB3_ = 0.29 V (nominal value).

Figure 6 shows a schematic of *G*_m2_ with the body-controlled DSL. The bias current of *G*_m2_ is 40 nA. The CMFB circuit (not shown) generates the output *V*_CMFB2_ using a 20 nA bias current (See Table 1 for the power consumed by the CMFB circuits). The overall current of *G*_m2_ is only 60 nA. Figure 7a shows a schematic of the DSL. The *R*_DSL1,2_ and *C*_DSL1,2_ are the resistors and capacitors in the DSL, respectively. *R*_DSL1,2_ is a variable pseudo-resistor controlled by *V*_PR_, which is realized by cascading floating PMOS transistors. The input of *G*_m3_ is associated with offset *V*_OS3_. Voltage *V*_OS3_ can disturb *V*_out_ of the CCIA similarly to other offsets (*V*_OS1_, *V*_OS2_, *V*_EOS,Gm2_). To reduce the effect of *V*_OS3_, two choppers, CH_D1_ and CH_D2_, are added to the integrator. Because the bandwidth of the integrator is relatively narrow (~30 mHz), *V*_OS3_ is up-modulated to the outside of the integrator’s bandwidth by CH_D2_. Figure 7b shows a schematic of the two-stage opamp for *G*_m3_. The first stage is biased using 5 nA. The second stage is biased at 200 nA for enhanced swing. The CMFB circuit generates *V*_CMFB3_ using a 5 nA bias current. The overall current is 210 nA.

The transfer function of the DSL has a low-pass characteristic for *V*_out_. It can be expressed as −*g*_mb1,2_/(s*R*_DSL1,2_*C*_DSL1,2_), where *g*_mb1,2_ is the body transconductance integrated into *G*_m2_. Within the feedback loop, the DSL creates a high-pass corner to reject *V*_EOS_. Using the condition *C*_fb1,2_ << *C*_in1,2_, the transfer function of the CCIA can be expressed as

(3) $$H(s)≅-\frac{A_{V1}g_{m1,2}}{C_{m1,2}}\frac{s}{\left(s+\eta \omega_{\mathrm{ugb}}/\beta A_{V1}\right)\left(s+\beta A_{V1}g_{m1,2}/C_{m1,2}\right)}$$

where *g*_m__1,2_ is the transconductance of the input pair of the *G*_m2_, *η* = (*g*_mb1,2_/*g*_m1,2_) ≈ 0.25, *ω*_ugb_ = 2*πf*_ugb_ = 1/(*R*_DSL1,2_*C*_DSL1,2_) is the unity-gain frequency of the integrator, and *β* = *C*_fb1,2_/*C*_in1,2_ is the feedback factor. Using (2), we obtain a high-pass corner frequency *f*_hp_ = (*η*/*βA*_V__1_) *f*_ugb_.

**Figure 8:**
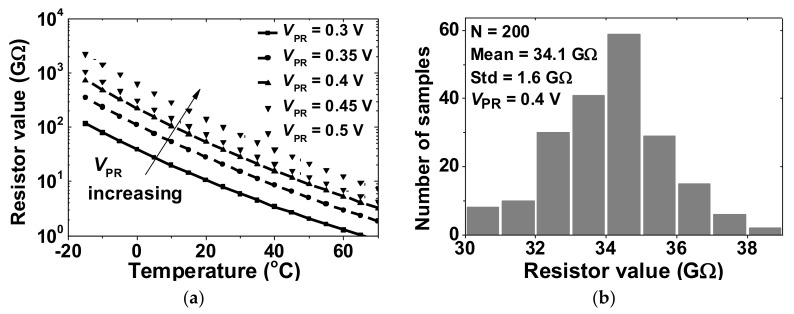
(**a**) simulated value of the pseudo-resistor as a function of temperature for various *V*_PR_ and (**b**) Monte Carlo simulation result of the pseudo resistor value at *V*_PR_ = 0.4 V.

**Figure 9:**
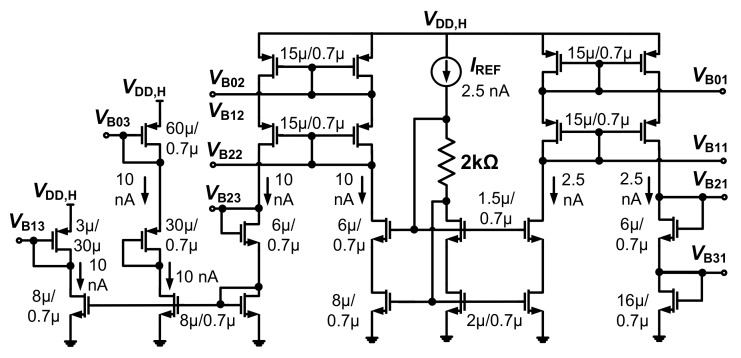
Schematic of the bias generator.

Because *f*_hp_ created by the DSL depends on the value of pseudo-resistor, we investigate the variability of *R*_DSL1,2_. Figure 8a shows the value of the *R*_DSL1,2_ as a function of temperature for various *V*_PR_. The resistance increases with *V*_PR_ while it decreases with temperature. Figure 8b shows the statistical distribution of the resistance obtained from Monte Carlo simulations at 27 °C and *V*_PR_ = 0.4 V. The result shows that the average value of *R*_DSL1,2_ is 34.1 GΩ with a standard deviation of 1.6 GΩ. Figure 9 shows a schematic of the bias generator. It consists of a constant-g_m_ current reference and six branches to generate the bias voltages for the amplifier. Overall current consumption is 47.5 nA.

**Figure 10:**
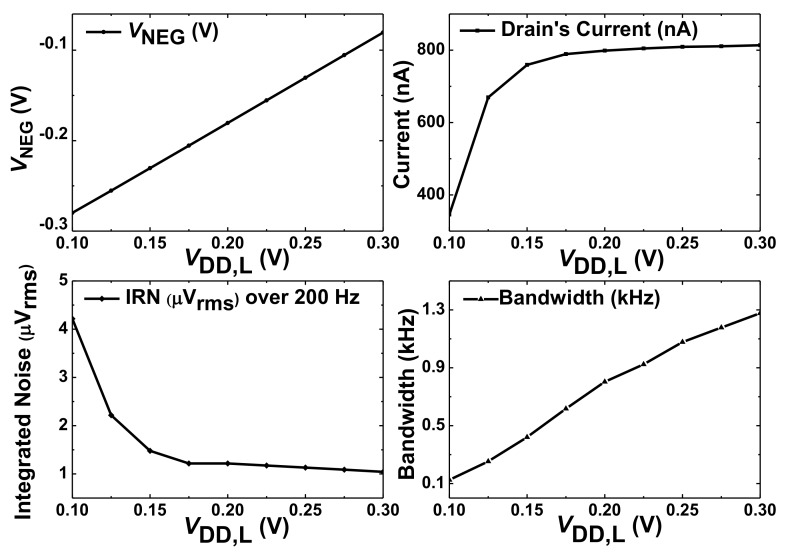
Simulated results showing the effect of *V*_DD,L_ on the noise and bandwidth of the proposed CCIA.

The proposed CCIA uses a narrow margin for the stacked transistors in the SQI stage. Therefore, we investigate the effect of supply and temperature on the performance of the amplifier. Figure 10 shows the effect of *V*_DD,L_ on the bias current (SQI stage only), noise, and bandwidth. When *V*_DD,L_ is increased, it is tracked by *V*_D_ and *V*_G_ in the negative generator, which increases *V*_NEG_ to keep the bias current. When *V*_DD,L_ is reduced below 0.15 V, the two stacked transistors are driven in the deep subthreshold region, which reduces the current and the gain. We note that the CCIA still operates with an integrated noise < 1.5 μV_rms_ when *V*_DD,L_ is reduced to 0.15 V. The amplifier bandwidth gradually increases with *V*_DD,L_, which agrees with the previous result [10].

Because *V*_DD,L_ is relatively low, an external electromagnetic interference can affect the sensor interface. In the proposed CCIA, the differential input signal *V*_IN_ is up-modulated to *f*_CH_ while the CM signal is not chopped. Therefore, chopping provides some means of rejection of external interference. In the case when the external interference exists at around *f*_CH_, it can affect the CCIA, however, this is well beyond the amplifier bandwidth (1–200 Hz). When the CCIA is used for the sensor readout, a theoretical input range calculated using a gain of 40 dB and the maximum output swing of 0.8 V_pp_ is 8 mV_pp_, which agrees with the measured value of 6 mV. Because the input is capacitively-coupled, it provides a relatively high DC blocking allowed by the voltage rating of *C*_in__1,2_.

**Figure 11:**
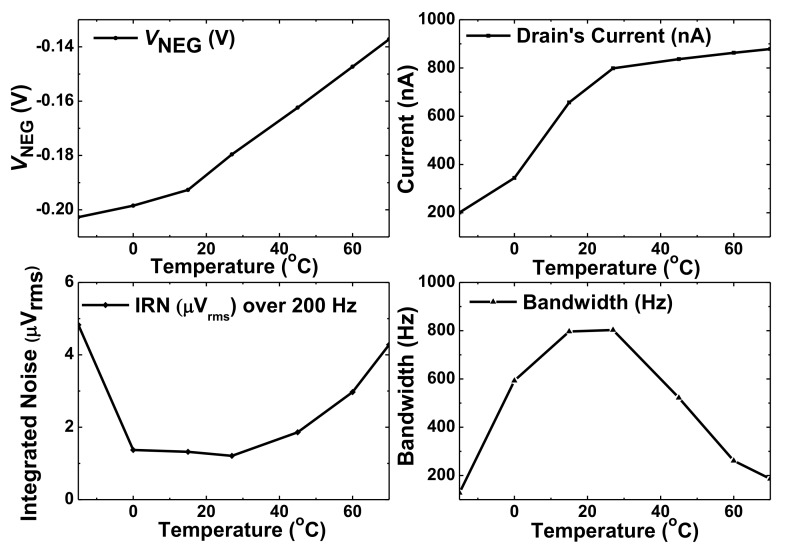
Simulated bias current, noise, and bandwidth depending on temperature.

Figure 11 shows the effect of temperature on the amplifier. The bias current increases with the temperature as expected from the constant-g_m_ current reference, which increases *V*_NEG_. The two temperature-dependent parameters of the subthreshold current are mobility and the threshold voltage [14]. The increased threshold voltage with temperature reduces the gain A_v_. The bandwidth can be expressed as *BW* = ω_p_(1+*β*A_v_), where ω_p_ is the 3-dB frequency and *β* is the feedback factor. Furthermore, the increased temperature reduces the bandwidth [15,16]. The amplifier achieves an integrated noise of less than 2 µV_rms_ over the temperature range from −5 °C to 45 °C.

The IRN of the CCIA, $\bar{V_{n,in}^{2}}$, can be expressed as

(4) $$\begin{array}{l} \bar{V_{n,\mathrm{in}}^{2}}={\left(\frac{C_{\mathrm{tot}}}{C_{\mathrm{in}1,2}}\right)}^{2}\left[\bar{V_{n,\mathrm{in},\mathrm{Gm}1}^{2}}+\frac{1}{A_{V1}}\left\{\bar{V_{n,\mathrm{in},\mathrm{Gm}2}^{2}}+\bar{V_{n,\mathrm{out},\mathrm{DSL}}^{2}}{\left(\frac{g_{\mathrm{mb}1,2}}{g_{m1,2}}\right)}^{2}\right\}\right]\\ ={\left(\frac{C_{\mathrm{tot}}}{C_{\mathrm{in}1,2}}\right)}^{2}\left[\frac{4kTnU_{\mathrm{th}}}{I_{\mathrm{DC}}}+\frac{8kTn}{A_{V1}g_{m1,2}}\left(1+\frac{g_{m3,4}+g_{m9,10}}{g_{m1,2}}\right)+\frac{2}{A_{V1}}{\left(\frac{g_{\mathrm{mb}1,2}}{g_{m1,2}}\right)}^{2}\left\{\left(8kTnR_{\mathrm{DSL}1,2}+\bar{V_{n,\mathrm{in},\mathrm{OTA}}^{2}}\right){\left(\frac{1}{sR_{\mathrm{DSL}1,2}C_{\mathrm{DSL}1,2}}\right)}^{2}\right\}\right] \end{array}$$

where C_tot_ = C_in1,2_ + C_fb1,2_ + C_p_, $\bar{V_{n,\mathrm{in},\mathrm{Gm}1}^{2}}$ and $\bar{V_{n,\mathrm{in},\mathrm{Gm}2}^{2}}$ are the input-referred noise of G_m1_ and G_m2_, respectively, $\bar{V_{n,\mathrm{out},\mathrm{DSL}}^{2}}$ is the output-referred noise of the DSL, and *g*_mi_ represents the transconductance of the transistors in *G*_m2_. The noise from the DSL includes the thermal noise of *R*_DSL1,2_ and the noise $\bar{V_{n,\mathrm{in},\mathrm{OTA}}^{2}}$ = 1.8 nV/√Hz of the two-stage opamp. We note that $\bar{V_{n,\mathrm{out},\mathrm{DSL}}^{2}}$ is not only multiplied by (*g*_mb1,2_/*g*_m1,2_)^2^ << 1, but is also reduced by *A*_V1_ = 29 dB. Using the values *g*_m1,2_ = 0.7 µS, *g*_m3,4_ = 0.35 µS, *g*_m9,10_ = 0.7 µS, *C*_in1,2_ = 4 pF, *C*_fb1,2_ = 40 fF, and *C*_p_ = 66.5 fF, we obtain $\bar{V_{n,in}^{2}}$ = 84.2 nV/√Hz. Using the shot noise model [10], we obtain a similar value for $\bar{V_{n,in}^{2}}$. Over the signal bandwidth of 200 Hz, the integrated noise contributions from *G*_m1_, *G*_m2_, DSL, and the other blocks are 44.9%, 39.1%, 12.5%, and 3.5%, respectively.

## 4. Measured Results

**Figure 12:**
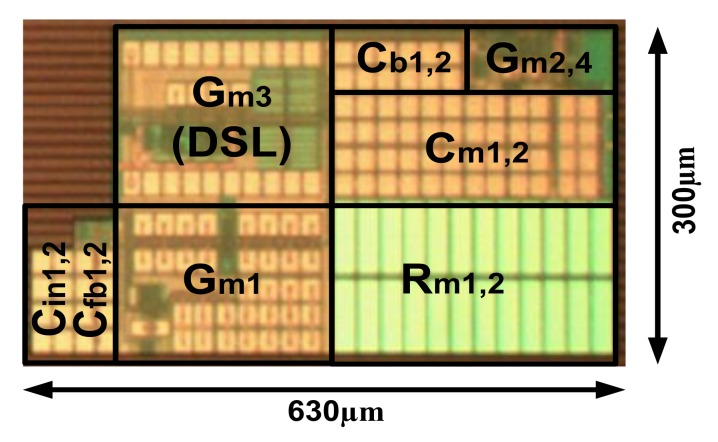
Chip microphotograph of the proposed CCIA.

**Figure 13:**
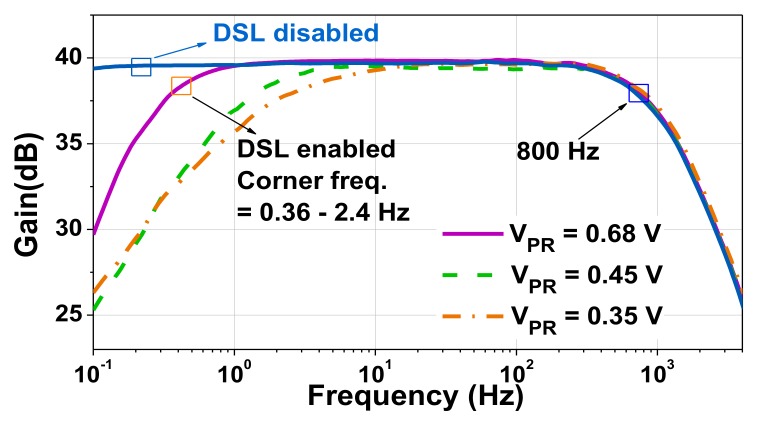
The measured frequency response.

**Figure 14:**
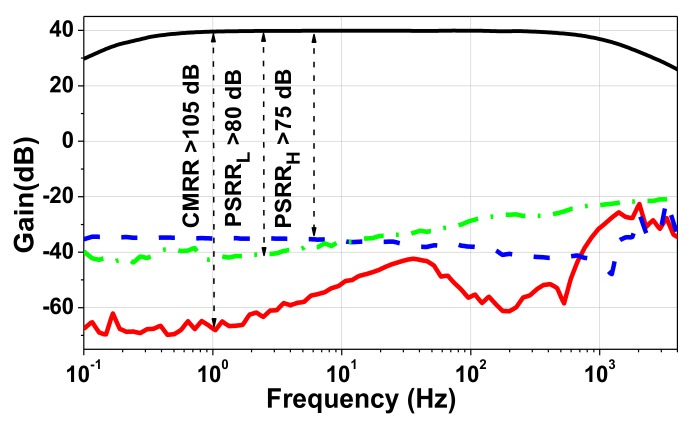
Measured CMRR and power supply rejection ratio (PSRR) as a function of frequency.

Figure 12 shows a microphotograph of the CCIA fabricated using a 180-nm CMOS process. The core area is 0.19 mm^2^. The supply voltages *V*_DD,L_ and *V*_DD,H_ are generated using external power supplies. Figure 13 shows the measured frequency response of the CCIA. The result shows a mid-band gain of 40 dB with a 3-dB bandwidth of 800 Hz. The high-pass corner *f*_hp_ was successfully created using the proposed DSL and varies from 0.36 to 2.4 Hz when *V*_PR_ is changed from 0.68 to 0.35 V. Figure 14 shows that the measured low-frequency CMRR > 105 dB. The power supply rejection ratios (PSRRs) measured at *V*_DD,L_ and *V*_DD,H_ show that low-frequency PSRR_L_ > 80 dB and PSRR_H_ > 75 dB, respectively.

**Figure 15:**
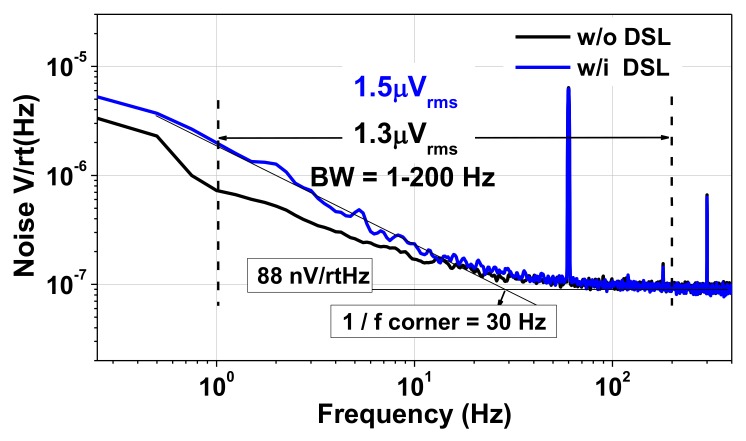
Measured input-referred noise voltage spectral density.

**Figure 16:**
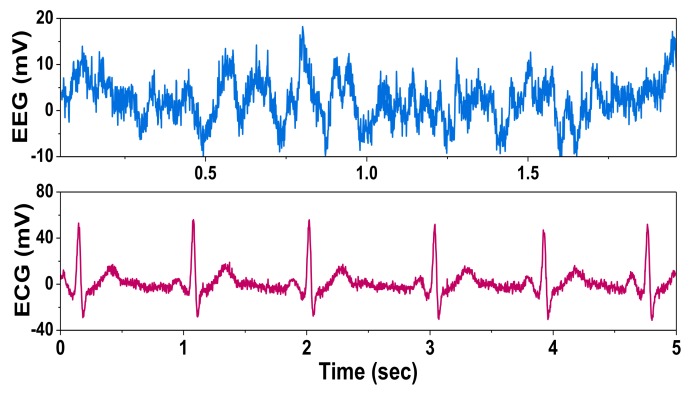
Measured output of the CCIA.

Figure 15 shows the measured noise spectral density. The input-referred noise density is 88 nV/rtHz, which is slightly higher than the calculated value of 84.2 nV/rtHz. When the DSL is enabled, the noise integrated from 1 to 200 Hz increases from 1.3 to 1.5 μV_rms_. We note that the noise contribution from the DSL is just 12.5%, which is much lower than the previous results of 89.5% [4] and 40.4% [6]. Figure 16 shows the measured output of the CCIA for prerecorded human EEG (~100 μV) and ECG (~1 mV) input signals [17]. Table 1 shows the power breakdown of the proposed CCIA.

Table 2 shows a performance comparison with the state of the art. The tradeoff between the noise and power can be evaluated using PEF as

(5) $$\mathrm{PEF}=V_{\mathrm{ni},\;\mathrm{rms}}^{2}\frac{2P_{\mathrm{DC}}}{\pi U_{\mathrm{th}}4kT\cdot BW}={\mathrm{NEF}}^{2}\cdot V_{\mathrm{DD}}$$

where *V*_ni,rms_ is the input-referred root-mean-square (rms) noise voltage, *P*_DC_ is the power consumption, and *BW* is the amplifier bandwidth. The previous approaches [4,7,12] use relatively-high currents to reduce noise. Because a high supply voltage *V*_DD_ > 1 V is used except for in [6], the large power consumption >1.8 µW leads to a relatively high PEF. By using the SQI stage with an ultra-low voltage, the proposed CCIA achieves a competitive noise performance of 1.5 μV_rms_ at a relatively low power of 0.61 µW (0.68 µW including bias generators). Our work achieves a good PEF of 10.2 (11.4 with bias generators) which is the lowest of the work shown in Table 2. Besides, the proposed CCIA has the lowest noise contribution of 12.5% from the DSL. The work in [10] achieves a good NEF/PEF = 2.1/1.6, however, their design does not include a DSL. Therefore, direct comparison is difficult. Although the dual power approach requires additional buck converter, a high-efficiency (>80%) converter consuming sub-nW can be used for voltage step-down [18,19].

## 5. Conclusions

In this paper, we investigated a sub-μW chopper amplifier using a noise-efficient DSL and power-efficient SQI stage. The proposed DSL not only removes the charge dividing effect but also reduces noise caused by both the transconductance ratio and the open-loop gain. Using the proposed approach, the noise contribution from the DSL is reduced to below 12.5%, which is much lower than the value seen in previous work. For power efficiency, we use an SQI stage biased by a supply voltage reduced to the 2*V*_DSAT_ saturation limit. The challenge of biasing the SQI stage and interfacing with a DSL having a different supply domain is addressed. Measurement of the fabricated CCIA shows an IRN of 1.5 μV_rms_ with the DSL enabled. The noise density is 88 nV/rtHz at a 40 dB gain when consuming 0.6 µW. The PEF is 11.4, which compares favorably with the state of the art.

## Figures and Tables

**Table 1:** Power breakdown.

Block	Components	Current (nA)	Voltage (V)
*G*_m1_ (SQI stage)	Input pair	1600	0.2
	CMFB	10	0.8
*G*_m2_ (Folded-cascode)	Input pair	20	0.8
	Cascode branch + CMFB	40	0.8
*G*_m3_ (Two-stage opamp)	Input pair	5	0.8
	Common source + CMFB	205	0.8
*G*_m4_ (Common-source)	Input pair	80	0.8
Bias circuits	Current mirror	80	0.2
	Bias generators	65.5	0.8
Total power	676.4 nW		

**Table 2:** Performance summary and comparison.

	[3]	[4]	[6]	[7]	[12]	This Work
Power (µW)	2.0	1.8	0.6	3.48	2.8	0.61/0.68 ^†^
Supply (V)	1.8	1.0	0.5	1.2	1.2	0.2/0.8
Current (µA)	1.1	1.8	1.2	2.9	2.3	1.6/0.36 1.68 ^†^/0.43 ^†^
Input cap. (pF)	15	12	12	20	1.0	4
Gain (dB)	41	40	40	40	25.7	40
CMRR (dB)	100	134	106	85	78	105
Noise (µV_rms_)	1.0	6.7	4.7	N/A	1.8	1.5
Noise floor (nV/rtHz)	100	60	140	47	80	88
Bandwidth (Hz)	100	100	250	N/A	200	200
DSL noise contribution (%)	N/A	89.5	40.4	26	N/A	12.5
NEF */PEF *	5.4/52.5	37.4/1398	7.5/27.9	3.9/18.3	7.4/66.4	5.7/10.2 5.9 ^†^/11.4 ^†^
Tech. (nm)	800	65	180	130	40	180
Area (mm^2^)	1.7	0.3	1.0	0.3	0.07	0.19

* Including DSL, **^†^** Including bias circuits. When the additional power (84 nW) of an 80% efficient buck converter is included, NEF/PEF increases to 6.5/12.6.
